# Efficient recycling of nutrients in modern and past hypersaline environments

**DOI:** 10.1038/s41598-019-40174-9

**Published:** 2019-03-06

**Authors:** Y. Isaji, H. Kawahata, N. O. Ogawa, J. Kuroda, T. Yoshimura, F. J. Jiménez-Espejo, A. Makabe, T. Shibuya, S. Lugli, A. Santulli, V. Manzi, M. Roveri, N. Ohkouchi

**Affiliations:** 1 Department of Biogeochemistry, Japan Agency for Marine-Earth Science and Technology (JAMSTEC), 2-15 Natsushima, Yokosuka, 237-0061 Japan; 2 Atmosphere and Ocean Research Institute, University of Tokyo, 5-1-5 Kashiwanoha, Kashiwa, Chiba 277-8564 Japan; 3 The Andalusian Earth Sciences Institute, The Spanish Research Council and the Univeristy of Granada, Avda de las Palmeras 4, 18100 Armilla, Spain; 4 Project Team for Development of New-generation Research Protocols for Submarine Resources, Japan Agency for Marine-Earth Science and Technology (JAMSTEC), 2-15 Natsushima, Yokosuka, 237-0061 Japan; 5 Department of Subsurface Geobiological Analysis and Research, Japan Agency for Marine-Earth Science and Technology (JAMSTEC), 2-15 Natsushima, Yokosuka, 237-0061 Japan; 6 Dipartimento di Scienze Chimiche e Geologiche, Università degli Studi di Modena e Reggio Emilia, Via Campi 103, 41125 Modena, Italy; 7 Istituto di Biologia Marina, Consorzio Universitario della Provincia di Trapani, Via Barlotta Giuseppe 4, 91100 Trapani, Italy; 8 Dipartimento di Scienze Chimiche, della Vita e della Sostenibilità Ambientale, University of Parma, Parco Area delle Scienze 157/A, 43124 Parma, Italy

## Abstract

The biogeochemistry of hypersaline environments is strongly influenced by changes in biological processes and physicochemical parameters. Although massive evaporation events have occurred repeatedly throughout Earth history, their biogeochemical cycles and global impact remain poorly understood. Here, we provide the first nitrogen isotopic data for nutrients and chloropigments from modern shallow hypersaline environments (solar salterns, Trapani, Italy) and apply the obtained insights to δ^15^N signatures of the Messinian salinity crisis (MSC) in the late Miocene. Concentrations and δ^15^N of chlorophyll *a*, bacteriochlorophyll *a*, nitrate, and ammonium in benthic microbial mats indicate that inhibition of nitrification suppresses denitrification and anammox, resulting in efficient ammonium recycling within the mats and high primary productivity. We also suggest that the release of ^15^N-depleted NH_3(gas)_ with increasing salinity enriches ammonium ^15^N in surface brine (≈34.0‰). Such elevated δ^15^N is also recorded in geoporphyrins isolated from sediments of the MSC peak (≈20‰), reflecting ammonium supply sufficient for sustaining phototrophic primary production. We propose that efficient nutrient supply combined with frequent bottom-water anoxia and capping of organic-rich sediments by evaporites of the Mediterranean MSC could have contributed to atmospheric CO_2_ reduction during the late Miocene.

## Introduction

Present-day and past hypersaline environments are unique and extremely important settings that affect biology, climate, and global geochemistry^1–3^. The biogeochemical cycle of hypersaline environments is highly distinct from that of freshwater and marine conditions because it is strongly affected by changes in the physicochemical parameters of the brine as well as in the state and composition of the biological community in response to salinity variations. In spite of the energy cost to overcome the salt stresses imposed on cellular systems^4^, hypersaline environments can be highly productive^5^. This is especially the case for shallow hypersaline environments, where densely populated microbial mats inhabited by a range of physiological groups of microorganisms are formed (e.g., cyanobacteria, phototrophic and chemotrophic sulfur-oxidizing bacteria, and sulfate-reducing bacteria). Constraining the biogeochemical cycle, which is responsible for the diversity of the niche space^6^ and the primary productivity^5,7^, is essential for understanding such ecosystems. Moreover, considering that hypersaline environments can become surprisingly productive, the massive evaporation events that occurred repeatedly throughout Earth’s history^8^ could have had a global impact. However, the biogeochemical cycles of the massive evaporation events remain poorly constrained.

Nitrogen has an active redox cycle comprised mainly of biological processes (e.g., nitrate and ammonium assimilation, N_2_-fixation, nitrification, denitrification, anaerobic ammonium oxidation [anammox]). It strongly influences photosynthetic primary productivity, and thus the entire biological community, by modifying the amount and chemical species of the bioavailable nitrogenous nutrients^9^. Because hypersaline microbial mats are often highly productive^5^, the nitrogen cycle in these systems is expected to operate such that the supply of bioavailable nitrogen is sufficient to maintain active photosynthesis. Various approaches have been applied to study nitrogen cycle, such as *in situ* measurements, culture and incubation experiments, and genetic analyses; some studies report the importance of N_2_-fixation^10,11^, whereas others have demonstrated that ammonium^10,12^ and dissolved organic nitrogen^13^ derived from the degradation of organic matter are crucial as nitrogen sources.

The nitrogen isotopic compositions (δ^15^N) of nitrogenous nutrients and organic compounds enable us to elucidate and quantify the relative importance of the processes controlling the flow of nitrogen^14,15^. Significantly, the isotopic signatures in modern hypersaline environments can be used for interpreting the δ^15^N signatures of the analogous ancient environments. In particular, the isotopic compositions of chloropigments (chlorophylls and bacteriochlorophylls) record primary signals of the physiology of phototrophs and the nitrogen substrates that they assimilate^16–18^. Moreover, the preservation of chloropigments as geoporphyrins in sediments on a geological timescale make them an ideal tool for reconstructing past nitrogen cycles^19–22^.

**Figure 1:**
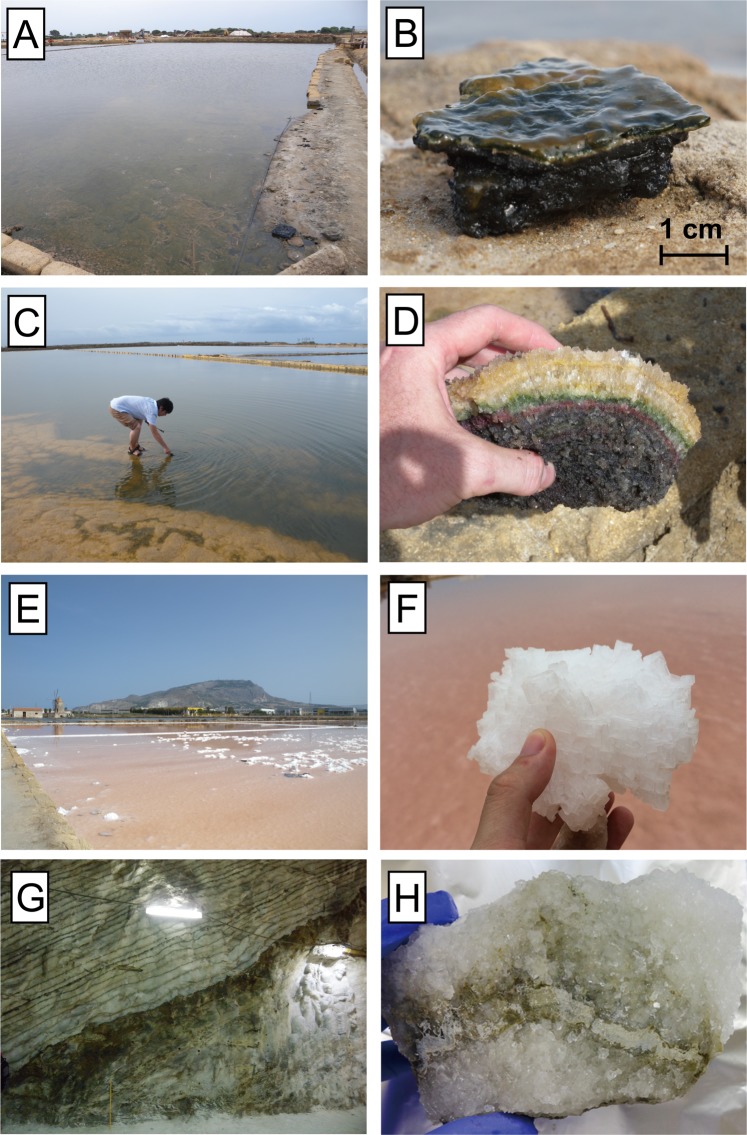
Description of samples analyzed. (**A**,**B**) Carbonate ponds are characterized by bottom deposits with a slimy layer several millimeters thick, composed of thin yellow, green, and pink layers on the surface and loose, black deposits underneath. (**C**,**D**) Gypsum ponds contain gypsum crusts with transparent yellowish, green, and pink layers (from the surface downward), and loose black deposits buried beneath. (**E**,**F**) Halite ponds are characterized by crusts of halite crystals several centimeters thick. (**G**,**H**) Halite–mud–anhydrite triplets of Unit C salts in the “Church section” of the Realmonte salt mine, and the rock sample analyzed. Modified from Isaji *et al*.^23^.

Here, we report the nitrogen cycle in a modern shallow hypersaline environment: the solar salterns in Sicily, Italy (Fig. 1A–F). The concentrations and nitrogen isotopic compositions of nitrate, ammonium, and chloropigments allowed us to trace the major processes occurring inside the microbial mats and surface brine. On the basis of insights obtained from modern solar salterns, we sought to reconstruct the nitrogen cycle during the peak of the massive evaporation event in the late Miocene, the Messinian salinity crisis, by measuring the nitrogen isotopic compositions of geoporphyrins from an ancient Sicilian deposit (Fig. 1G,H).

## Distributions and δ^15^N Signatures of Nitrogenous Compounds

We investigated three commercial solar salterns at Trapani, western Sicily, Italy: the Sosalt (SS), Culcasi (CU), and Chiusicella (CH) salterns (Fig. S1). Three saltern pond types can be defined according to the evaporite minerals that progressively precipitate at the bottom: calcium carbonate (CaCO_3_) and gypsum (CaSO_4_·2H_2_O) ponds, where benthic microbial mats are present, and halite (NaCl) ponds, which lack a benthic ecosystem (Fig. 1A–F). The degree of evaporation of the surface brine in each pond is calculated from the molar concentrations of Mg^2+^ in the seawater and brine samples as follows (Table S1):

1 $${{\rm{DE}}}_{{\rm{Mg}}}=\frac{[{{\rm{Mg}}}_{{\rm{brine}}}^{2+}]}{{[\mathrm{Mg}}_{{\rm{seawater}}}^{2+}]}$$

**Figure 2:**
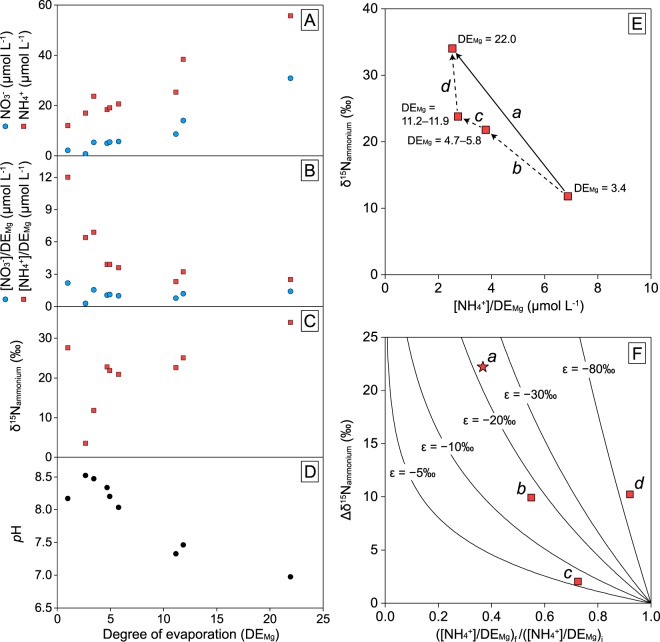
Variations in the concentrations and δ^15^N values of nitrate and ammonium in the Trapani solar salterns. (**A**) Concentrations of surface-brine nitrate (blue circles) and ammonium (red squares) along the salinity gradient in the samples collected in 2015, (**B**) surface-brine nitrate and ammonium concentrations normalized to the degree of evaporation (DE_Mg_), (**C**) δ^15^N of the surface-brine ammonium, and (**D**) pH^23^. (**E**) Cross plot of δ^15^N and [NH_4_^+^]/DE_Mg_ of the surface-brine ammonium. Ponds with similar DE_Mg_ values are averaged. Arrow *a* averages the entire evaporation path from the carbonate to the halite pond, and arrows *b* to *d* represent more detailed evaporation paths through each pond type. (**F**) Changes in the δ^15^N plotted against normalized changes in concentration of ammonium through the evaporation paths indicated in the arrows in (**E**). Lines represent expected pathways of isotopic composition assuming the Rayleigh distillation model for the different fractionation factors shown. The ε calculated for arrow *d* is probably an artifact derived from the variability of the ammonium concentrations in the different salterns.

Normalization of the solute concentrations in the surface brine by using DE_Mg_ cancelled out the effect of the condensation of solutes during evaporation and thereby allowed us to examine the addition or removal of the solutes (Fig. 2A,B).

**Figure 3:**
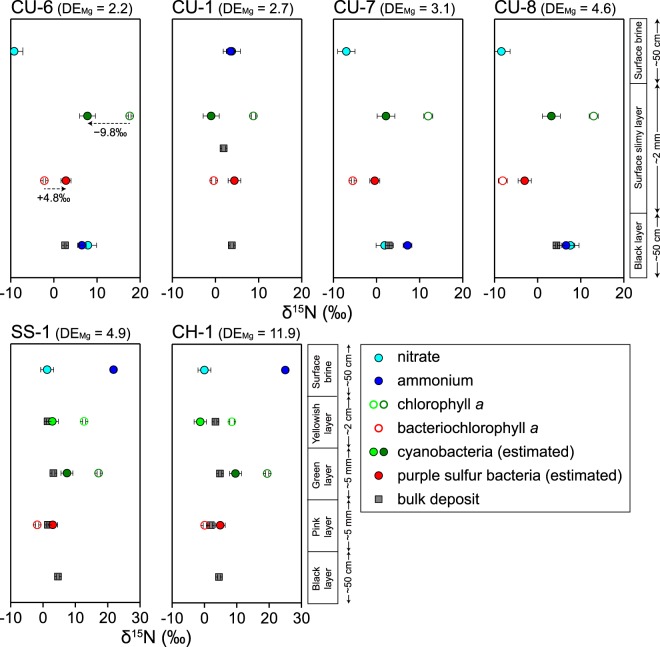
Depth profiles of δ^15^N in the microbial mats in the carbonate ponds (CU-1, -6, -7, -8) and the gypsum crusts in the gypsum ponds (SS-1, CH-1). The δ^15^N values of cyanobacteria (solid light- and dark-green circles) were calculated from chlorophyll *a* (open light- and dark-green circles), and those of the purple sulfur bacteria (solid red circles) from bacteriochlorophyll *a* (open red circles). Light- and dark-blue circles indicate δ^15^N of nitrate and ammonium, respectively, in the surface brine and porewater. Gray squares indicate δ^15^N values of total organic nitrogen. Also shown are rough estimates of the thickness of each layer. CU, Culcasi; SS, Sosalt; CH, Chiusicella.

All microbial mats and gypsum crusts investigated had similar distributions of chloropigments, which are mostly derived from benthic rather than planktic phototrophs^23^. We determined δ^15^N values for the chloropigments derived from the main phototrophs inhabiting each layer of the microbial mats: chlorophyll *a* from cyanobacteria, which generally dominate the yellow and green layers, and bacteriochlorophyll *a* from the purple sulfur bacteria living in the pink layers^24^. In contrast to the bulk δ^15^N values that varied over a range from 0.5‰ to 4.8‰, those of chloropigments differed dynamically, with higher values for chlorophyll *a* (8.8‰ to 19.4‰) than for bacteriochlorophyll *a* (−8.1‰ to 0.1‰) in all ponds (Fig. 3, Table S2).

To investigate the physiology of phototrophs and their role in the nitrogen cycle, the δ^15^N values of phototrophs must be estimated from those of the chloropigments. Laboratory culture experiments and field observations have yielded several relationships between the δ^15^N of chlorophyll *a* and cyanobacteria: ^15^N enrichment of 9.8‰ ± 1.8‰^25,26^, ^15^N depletion of 0.9‰ ± 1.3‰^25^, and ^15^N depletion of 4.8‰ ± 1.4‰^27^ in chlorophyll *a* relative to the cell. Here, we assumed 9.8‰ ± 1.8‰ ^15^N enrichment in chlorophyll *a* relative to the cell^25^, because the δ^15^N values of cyanobacteria estimated from the other relationships differed unrealistically from those of the bulk deposits (Table S2). On the other hand, the δ^15^N relationship between purple sulfur bacteria and their bacteriochlorophyll *a* is considered to be similar to that of the eukaryotic algae and their chlorophyll *a*^25^. Thus, we applied an ^15^N depletion of 4.8‰ ± 1.4‰ in bacteriochlorophyll *a* relative to the cell^20,27^. The resulting variations of δ^15^N values with depth in the phototrophs show similar trends in all ponds, with cyanobacteria in the green layers enriched in ^15^N relative to purple sulfur bacteria, except for pond CU-1 (Fig. 3).

The concentrations and δ^15^N patterns of nitrate and ammonium (NH_4_^+^ and NH_3(aq)_) also showed similar depth profiles in the studied ponds. Ammonium was the largest pool of dissolved inorganic nitrogen (334.3–558.7 µmol L^−1^); its concentrations were more than an order of magnitude higher than those of the surface-brine nitrate, ammonium, and porewater nitrate (Table S1). The porewater ammonium pools had a narrow δ^15^N range, between 6.5‰ and 7.2‰, whereas ammonium in the surface brine was more enriched in ^15^N and showed a progressive increase along the salinity gradient, to as high as 34.0‰ in the halite ponds (Fig. 2C). Although the concentrations and δ^15^N values of nitrate and ammonium in the surface brine varied interannually (Table S2), the similar depth variations of δ^15^N values of the phototrophs suggest that similar chemical conditions and similar biological processes occurred inside the mats. Variability in the surface brine has also been described previously^12^ and possibly reflect climate conditions during the previous several days to weeks, such as light availability and rainfall.

## Nitrogen dynamics in microbial mats

The similar δ^15^N values for porewater ammonium and black layer deposits indicate that the ammonium was supplied by anaerobic degradation of organic matter in the black anoxic layer. The general decrease of ammonium concentrations toward the top of the mat^10,11^ suggests net upward diffusion and consumption of ammonium by processes such as ammonium assimilation, nitrification, or anammox.

The consumption of porewater ammonium by nitrification is of minor importance, considering the low nitrate concentrations (Table S1). Immediate utilization of the produced nitrite or nitrate could keep the concentrations low, but the relatively low δ^15^N of porewater nitrate suggests that assimilation, denitrification, and anammox, which fractionate against ^15^N-enriched nitrate^14,28^, are inactive. Because denitrification and anammox are the major pathways for removing bioavailable nitrogen from the system, their suppression results in accumulation of bioavailable nitrogen within the mat. The cause of the suppression of nitrification is attributable to the extreme energy cost of the hypersaline conditions^4^, photoinhibition^29^, or the presence of phototrophic bacteria, which out-compete nitrifying bacteria and archaea for ammonium^30^.

**Figure 4:**
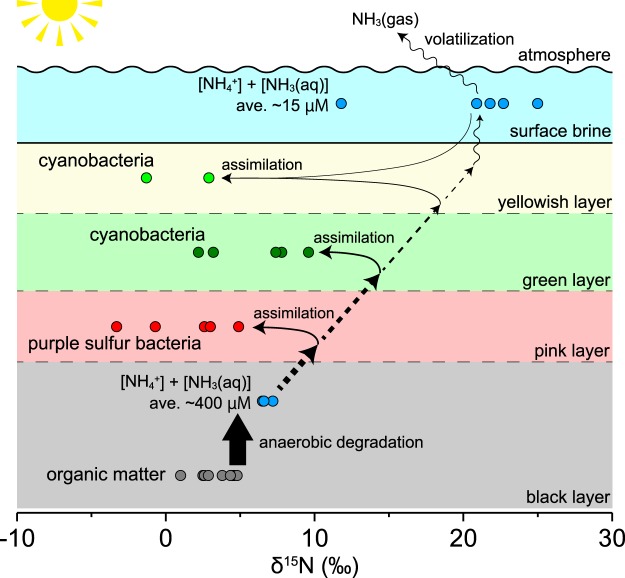
Proposed nitrogen cycle for the carbonate and gypsum ponds in the Trapani solar salterns. Ammonium (blue circles) produced by the anaerobic degradation of organic matter (grey circles) is assimilated by purple sulfur bacteria (red circles) and cyanobacteria (green and light-green circles) as it diffuses upward, leaving behind substantially ^15^N-enriched ammonium, of which some part possibly leaks into the surface brine. The increase in salinity causes the release of ^15^N-depleted NH_3(gas)_ to the atmosphere, thereby increasing the δ^15^N of the surface-brine ammonium.

The δ^15^N values of the phototrophs indeed support the possibility of ammonium assimilation as a major consumption pathway. The δ^15^N values of purple sulfur bacteria cover a range where both the porewater nitrate and ammonium could be their nitrogen source. However, the low nitrate concentrations in the porewater, at only about 1/300 of the ammonium concentration (Table S1), as well as a general preference for ammonium over nitrate as a substrate^31^, suggest ammonium as their major nitrogen source. The assimilation of porewater ammonium by purple sulfur bacteria leaves ^15^N-enriched ammonium, which diffuses upwards to be assimilated by cyanobacteria in the green and yellow layers, resulting in distinctly higher δ^15^N values in cyanobacteria than in purple sulfur bacteria (Fig. 4). The lower δ^15^N of cyanobacteria in the yellowish transparent layer compared with that in the green layer in the gypsum crusts could be attributable to lower primary productivity in the former layer, which has lower chlorophyll *a* concentrations by at least an order of magnitude.

Among several mechanisms for ammonium transport through the cell membrane and subsequent biosynthetic pathways, the phototrophs in the mats actively transport NH_4_^+^ and assimilate it with glutamine synthase in the range of porewater ammonium concentrations found in this study, with associated nitrogen isotopic fractionation (ε) ranging from −4‰ to −20‰ in laboratory culture experiments under freshwater to marine conditions^32–34^. Our rough estimates of ε for phototrophic assimilation of ammonium inside microbial mats show ε values for purple sulfur bacteria (ε_PSB_) of −10‰ to −5‰ for CU-6, −15‰ to −10‰ for CU-7, and −18‰ to −12‰ for CU-8 (Fig. S2). Although we could not constrain ε for cyanobacteria because of its strong dependence on the assumed ε_PSB_, our results imply that the range of ε for phototrophic assimilation of ammonium under hypersaline conditions is consistent with that reported for freshwater to marine conditions^32–34^.

The δ^15^N values of chloropigments therefore suggest that the major source of nitrogen for phototrophs is ammonium. However, the large discrepancies of some samples between bulk δ^15^N values and the estimated cyanobacterial δ^15^N (SS-1 and CH-1; Table S2) indicate the presence of non-phototrophic biomass depleted in ^15^N. A possible candidate for this biomass is diazotrophic microorganisms, which have δ^15^N ranging between −2‰ and 0‰^35^. This is consistent with previous incubation experiments and genetic analyses indicating N_2_-fixation in hypersaline microbial mats. In particular, our results are consistent with the suggestion that the activity of non-phototrophic microorganisms such as chemoautotrophic or heterotrophic N_2_-fixers is important^11,36,37^. The abundant organic matter produced by photosynthesis probably supports the extreme energy expenditure of these diazotrophs on N_2_-fixation and adaptation to hypersaline conditions. Thus, the high primary productivity of the hypersaline microbial mats is supported by efficient recycling of ammonium within the mat in the absence of nitrification, denitrification, or anammox, as well as by the supply of new nitrogen from chemoautotrophic and/or heterotrophic diazotrophs.

## Ammonium ^15^N enrichment under evaporative conditions

The δ^15^N of ammonium in the surface brine increased progressively along the salinity gradient (Fig. 2C). This could be due in part to the leakage of ^15^N-enriched ammonium from the mat, produced as a result of the assimilation of ammonium by benthic phototrophs. However, considering the net decrease in surface-brine ammonium with increasing salinity ([NH_4_^+^/DE_Mg_]; Fig. 2B), there are likely other processes consuming ^15^N-depleted ammonium. Here, we estimated an apparent nitrogen isotopic fractionation factor assuming that the surface-brine ammonium in all investigated ponds behaved as a continuous system. The overall ε for the entire succession from the carbonate (CU-2) to the halite pond (CU-5) was around −22‰, with ε in the carbonate and gypsum ponds ranging from −5‰ to −20‰ (Fig. 2E,F). Although the extremely high ε calculated for the halite pond is probably an artifact of the variability of the ammonium concentrations in the different salterns, it seems likely that ^15^N-depleted ammonium was removed even after the saturation point of halite.

We suggest that the δ^15^N increase in surface-brine ammonium can be ascribed to the release of NH_3(gas)_ to the atmosphere due to the decrease in gas solubility along the salinity gradient. A laboratory study has suggested that the release of NH_3(gas)_ from fluids occurs episodically; between these release episodes the gas stays in the fluids and maintains isotopic equilibrium, with NH_3(aq)_ depleted in ^15^N relative to NH_4_^+^ by 45.4‰ at 23 °C^38^. Although the pH decline from 8.5 in carbonate ponds to 7.0 in halite ponds (Fig. 2D) is associated with a decrease in the relative proportion of total ammonium as NH_3(aq)_ from around 10% to less than 1%, continuous evaporation, inducing repeated degassing and subsequent dissociation under isotopic equilibrium, could result in a progressive δ^15^N increase in the remaining ammonium. We expect that ^15^N-enriched ammonium can be used as an indicator to constrain the nitrogen cycle of hypersaline environments, where ammonium is abundant and partially dissociates to NH_3(aq)_.

## Nitrogen cycle during the MSC peak

During the late Miocene (5.97–5.33 Ma), the Mediterranean Sea experienced a massive evaporation event referred to as the Messinian salinity crisis (MSC^39,40^). Its peak occurred during a short time window (stage 2; 5.60–5.55 Ma^40^) and was accompanied with the precipitation of huge volumes of primary halite in the deep Mediterranean settings. Here, we measured the δ^15^N of geoporphyrins purified from the mud–anhydrite layers between the halite layers of the Realmonte salt mine (Sicily, Italy), which were deposited in the Caltanissetta basin during the MSC peak (Fig. 1G,H). These layers are considered to have been deposited under density-stratified conditions during humid seasons of the year, formed as a result of the continental inflow that capped the relatively shallow brine water-body, which precipitated halite during the arid seasons^41^. The lack of a mat structure in the mud layer suggests that the phototrophs inhabited the water column rather than the bottom deposits.

**Figure 5:**
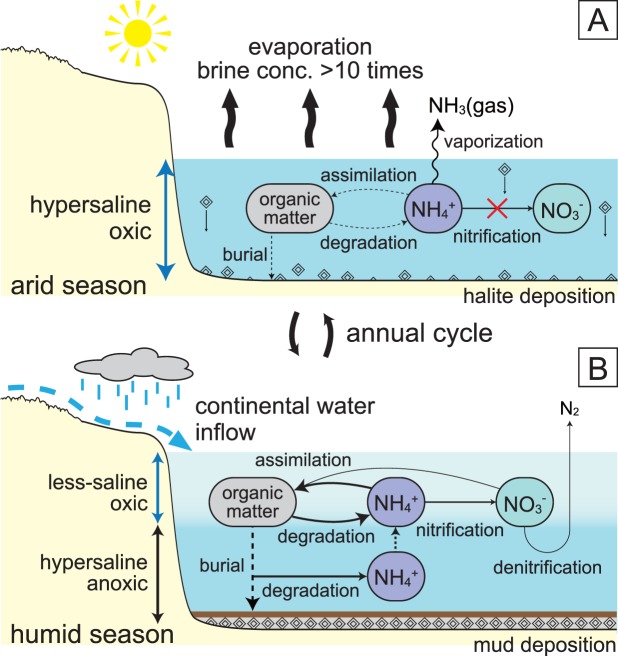
Proposed nitrogen cycle during the peak of the Messinian salinity crisis. (**A**) Nitrogen cycle in the arid season during the deposition of Unit C halite of the Realmonte salt mine. The suppression of nitrification and degassing of ^15^N-depleted NH_3(gas)_ resulted in the accumulation of ^15^N-enriched ammonium in the brine. (**B**) Nitrogen cycle in the humid season during the deposition of the mud layer. ^15^N-enriched ammonium accumulated in the subsurface was assimilated by phototrophs, resulting in the elevated δ^15^N of the geoporphyrins.

Two mud–anhydrite-layer samples had identical Fe-porphyrin compositions (Fig. S3). The δ^15^N values for the combined geoporphyrins from each sample were 17.2‰ and 21.7‰ (Table S2), which we interpret to reflect the assimilation of ^15^N-enriched ammonium pools. We propose that the ^15^N-enriched ammonium was produced by mechanisms similar to those discovered for the solar salterns: the suppression of nitrification under extreme salinity and the release of NH_3(gas)_ during the arid season (Fig. 5). The supply of this ^15^N-enriched ammonium to the less-saline surface layer during the following humid season resulted in ^15^N-enriched phototrophic biomass. During the humid season, nitrification in the less-saline surface layer and the associated denitrification may have also contributed to increased δ^15^N of nitrate and ammonium^42^. However, because subsurface ammonium is the starting material for these processes, its ^15^N-enrichment during the arid season should have acted to elevate the overall δ^15^N of the nitrogenous compounds in the system.

The first insights into the nitrogen cycle of the MSC provided by our study suggests that ammonium accumulated in the subsurface anoxic brine was supplied to the surface phototrophs under density-stratified conditions at a rate sufficient to sustain phototrophic primary production (Fig. 5). It is reasonable to believe that other essential nutrients, such as phosphate, were also supplied from the subsurface to enhance the phototrophic primary productivity. Importantly, the preservation efficiency of the organic matter produced must have been increased because of the presence of the anoxic bottom brine and the capping of every shale layer by halites. Stratigraphic and geochemical investigations suggest that such an efficient carbon sequestration may actually have been a common feature of the MSC. For example, organic-rich shales, marls, and dolomites have been found widespread and repeatedly throughout the MSC sequence^43,44^, and there are descriptions of rapidly growing gypsum crystals effectively trapping filamentous organic material^45,46^. It is noteworthy that about 50% of the world’s largest oilfields are sealed by evaporites, although they constitute less than 2% of the world’s sedimentary rocks^47^.

The role of the Mediterranean MSC as a carbon sink needs to be explored because it has important implications for the remarkable shifts in the global carbon cycle during the late Miocene. This period is characterized by a decline in the atmospheric CO_2_ level, which occurred concurrently with intense tectonic activity and expansion of the Antarctic ice sheet^48^. The major decrease in the CO_2_ concentration, together with other regional factors, may have promoted substantial and irreversible changes in ecosystems from 7 to 5 Ma, such as the global expansion of C4 plants^49,50^ and modifications in carbon acquisition strategies of marine algae^51^. The extensive salt formations and the efficient organic carbon burial could also have contributed to the atmospheric CO_2_ decrease. Nevertheless, a precise, quantitative evaluation is still difficult at this stage because the MSC intermediate and deep basin deposits remain virtually unexplored. Future drilling of cores from the deep Mediterranean basin (e.g., MEDSALT initiative, https://medsalt.eu) would reveal whether efficient carbon sequestration in marginal basins is a local process or widespread in the Mediterranean, thereby clarifying the impact of the MSC on the global carbon cycle.

## Conclusions

Our study highlights the dynamic variations of the nitrogen isotopic signatures in solar salterns, suggesting that the high primary productivity of the hypersaline microbial mats is supported by efficient recycling of ammonium resulting from the suppression of nitrification, denitrification, and anammox. This first report of the highly distinct δ^15^N signatures of chloropigments in the benthic microbial mats is expected to be important comparison data for elucidating the nitrogen cycle of ancient analogous environments such as the stromatolites, which strongly influenced Earth’s surface biogeochemistry^3^. Moreover, the substantially high δ^15^N of ammonium formed during evaporation is useful in clarifying the nitrogen cycle of hypersaline environments: we interpret the extremely high δ^15^N of geoporphyrins from the MSC peak in the Caltanissetta basin as reflecting a supply of ammonium from the subsurface anoxic brine sufficient to sustain phototrophic primary production. In combination with the frequent formation of bottom water anoxia and capping of organic-rich sediments by evaporites, our first insights into the nitrogen cycle during the MSC imply efficient carbon sequestration, which might have contributed to the decrease in atmospheric CO_2_ during the late Miocene.

## Materials and Methods

### Trapani solar salterns

Three commercial solar salterns at Trapani (western Sicily, Italy) were investigated: the Sosalt (SS), Culcasi (CU), and Chiusicella (CH) salterns (Fig. S1). These salterns, each consisting of multiple ponds with different salinities, differ in scale. Sosalt is the largest and Chiusicella is the smallest in both surface area and the number of ponds.

Three pond types can be defined in the solar salterns, according to the evaporite minerals that precipitate at their bottoms: calcium carbonate (CaCO_3_) ponds, gypsum (CaSO_4_·2H_2_O) ponds, and halite (NaCl) ponds. The carbonate ponds (salinity <150), in which calcium carbonate precipitates, are characterized by the formation of a dense benthic microbial mat (Fig. 1A,B) that consists of a slimy layer a few millimeters thick, which is composed of thin yellow, green, and pink layers on the surface, and black, loose deposits buried underneath. In the gypsum ponds (salinity 150–350), a thick layer of selenite gypsum crust grows up from the bottom (Fig. 1C,D), forming layers of different colors (yellowish transparent, green, and pink) from the surface to an average depth of ~5 cm, with loose black deposits below these. Large halite crystals form in the halite ponds (salinity > 350), in which benthic microbial communities are apparently absent (Fig. 1E,F).

Normal seawater, surface brine, porewater, and bottom deposits were collected from the ponds during the daytime in September 2015 and 2016 (Table S1). The surface brine samples and bottom deposits were collected from six carbonate ponds (SS-3, CU-1, -2, -6, -7, and -8), three gypsum ponds (SS-1, SS-2, and CH-1), and two halite ponds (SS-4 and CU-5). The brine samples were immediately passed through 0.45 µm filters to minimalize any biological activity before the nitrate and ammonium concentrations and their nitrogen isotopic compositions were measured. We also collected porewater from three carbonate ponds (CU-6, -7, and -8). Sediment cores, ~50 cm in length, were recovered from each pond, and a Rhizon sampler connected to a 0.45 µm filter was inserted into the black layer for sample collection, at 2.5 cm below the deposit surface in CU-6 and at 5 cm in CU-7 and CU-8. All samples were stored in a freezer until analysis.

### Concentrations and nitrogen isotopic compositions of NO_3_^−^ and NH_4_^+^

Our experimental procedures largely followed those reported previously^52,53^. The concentration and nitrogen isotopic composition of NO_3_^−^ were measured with the microbial denitrifier method^54^. *Pseudomonas chlororaphis* subsp. *aureofaciens* ATCC 13985 ^T^, a denitrifying bacterium lacking the capacity to reduce N_2_O^55^, was used to convert NO_3_^−^ to N_2_O in a glass vial. The N_2_O produced was purged from the vial with ultrapure helium and trapped cryogenically with liquid nitrogen (PreCon, Thermo Finnigan, Bremen, Germany). The purged N_2_O was then introduced into a gas chromatograph (Agilent 6890, Agilent Technologies, Santa Clara, California, USA) equipped with a PoraPLOT column (25 m × 0.32 mm, Agilent Technologies) and a GC III Interface (Thermo Finnigan) to purify the N_2_O. The nitrogen isotopic composition of the purified N_2_O was measured with an isotope ratio mass spectrometer (IRMS: Finnigan DELTAplus XP, Thermo Finnigan). The isotopic data was calibrated with three isotopic nitrate standards (USGS32, USGS34, and IAEA-NO_3_). The isotopic compositions are expressed in conventional δ notation relative to AIR. The analytical precision was within 2.0‰ (±1σ).

We used the diffusion method to recover the total ammonium (NH_4_^+^ and NH_3_(aq)) as (NH_4_)_2_SO_4_^2−^ ^52,56^. The (NH_4_)_2_SO_4_^2−^ was then converted to NO_3_^−^ with the persulfate oxidation method^52,57^. The nitrogen isotopic composition of the NO_3_^−^ produced was measured with the microbial denitrifier method. The measured δ^15^N of N_2_O was calibrated to that of total ammonium using three ammonium isotopic standards (IAEA-N2, USGS25, and USGS26) that were analyzed concurrently with the samples. The analytical precision was within 1.0‰ (±1σ). The concentration of total ammonium was measured with a fluorometric or colorimetric technique; i.e., the o-phthalaldehyde method^58^ for the surface brine of CU-6, -7, and -8, and the indophenol blue method^59^ for all other samples.

### Compound-specific nitrogen isotopic compositions of chloropigments

For the bulk δ^15^N measurements, the microbial mat samples were separated into two parts: the upper slimy layer and the underlying loose black deposits, and the gypsum crusts were separated into four parts: the yellowish transparent, green, and pink gypsum layers, and the underlying loose black deposits. The subsampled deposits were freeze-dried, powdered, and treated with 0.1 M HCl to remove CaCO_3_ in precleaned smooth-wall tin capsules before analysis. The δ^15^N measurements were conducted using a modified Flash EA1112 automatic elemental analyzer (EA) connected to a Thermo Finnigan Delta plus XP IRMS via a ConFlo III Interface^60^. Based on repeated measurements of our laboratory standards, the analytical error was estimated to be within 0.6‰ (±1σ).

The analytical procedure used for the purification of chloropigments followed that described previously^23^. In brief, the surface deposits of the microbial mats (CU-1, -6, -7, and -8), and the yellowish transparent, green, and pink layers of the gypsum crusts (SS-1 and CH-1) were each freeze-dried and ground to powder. The samples were extracted three times with acetone by sonication, and then extracted with *n*-hexane. The *n*-hexane fraction was dried completely under N_2_ gas and dissolved in 100 µL of *N*,*N*-dimethylformamide for HPLC analysis. All procedures were performed in a dark room.

The pigments were isolated and purified with dual-step HPLC using an Agilent Zorbax Eclipse XDB-C18 column (4.6 mm × 250 mm; 5 μm silica particle size). The pigments were eluted isocratically with 75% acetonitrile: pyridine (100:0.5, v/v) and 25% ethyl acetate: pyridine (100:0.5, v/v) for 5 min, followed by a linear gradient of ethyl acetate: pyridine to 50% over 50 min. The flow rate was set to 1 mL min^−1^ and the column temperature to 30 °C. The collected chlorophyll *a* and bacteriochlorophyll *a* were dissolved in 1.5 mL of hexane and reacted with 2 M HCl to convert them to pheophytin *a* and bacteriopheophytin *a*, respectively for the second purification step with HPLC. The column used for the second purification step was an Agilent Zorbax Eclipse PAH column (4.6 mm × 250 mm; 5 μm particle size). The pigments were eluted isocratically with 80% acetonitrile: pyridine (100:0.5, v/v) and 20% ethyl acetate: pyridine (100:0.5, v/v) for 5 min, followed by a linear gradient of ethyl acetate: pyridine to 60% over 25 min, and a linear gradient of ethyl acetate: pyridine to 100% over 10 min. The flow rate was set to 1 mL min^−1^ and the column temperature to 15 °C.

The stable nitrogen isotopic compositions of the chloropigments were measured using a modified EA/IRMS^59^. The purified chloropigments were dissolved in dichloromethane, transferred to precleaned smooth-wall tin capsules, and dried before analysis. Based on repeated measurements of our laboratory standards, the analytical error was estimated to be within 0.7‰ (±1σ).

### Realmonte salt mine

The Realmonte salt mine is composed of a salt unit in the Caltanissetta Basin in Sicily (Fig. S1), deposited during the peak (stage 2) of the MSC at 5.60–5.55 Ma^61,62^. The salt unit has been divided into four main lithological units from base to top: A–D^63,64^. Unit C, investigated in this study, was deposited after the basin had undergone desiccation^64^ and is composed of more than 120 lithological cycles, each one represented by the superposition of three facies: (i) a millimeter-thick mud layer; (ii) a millimeter–centimeter-thick anhydrite (CaSO_4_) or polyhalite (K_2_MgCa_2_(SO_4_)_4_·2H_2_O) layer; and (iii) a decimeter-thick halite layer with white patchy polyhalite grains (Fig. 1G)^64,65^. The highly pure halite cumulates of skeletal hoppers with chevron overgrowths indicate that the precipitation occurred in a relatively shallow, nonstratified water body, whereas the mud layers are considered to have been deposited under a bottom-anoxic stratified water body^41,64^. The triplets are interpreted as reflecting annual cyclicity, with anhydrite–halite layers representing the dry season and the mud layers the wet season with increased terrestrial runoff^41^.

Fallen rocks consisting of a mud–anhydrite layer sandwiched between halite layers (Fig. 1H) were collected at “Church section” (approximately at 10^th^ cycle) and “Rosone section” (towards the bottom of Unit C)^41^, where Unit C mud–anhydrite–halite triplets are observed. Before the analysis, the halite layer of the rock sample was largely removed with a chisel and hammer. The remaining mud–anhydrite layer of the rock sample was washed in Milli-Q water, methanol, and dichloromethane for 1 min each with sonication to eliminate any possible contamination on the surface of the rock.

### Compound-specific isotopic compositions of geoporphyrins

The analytical procedure for the analysis of geoporphyrins was modified from a previously reported procedure^66^. The geoporphyrins contained in the pulverized sediment samples were extracted with dichloromethane: methanol (7:3, v/v). The extract was then separated with silica gel column chromatography and the low-polarity fraction was collected with dichloromethane. This was further separated into six subfractions with another round of silica gel column chromatography; the reddish-colored band was collected with dichloromethane: methanol (95:5, v/v).

The individual porphyrins were isolated and purified with dual-step HPLC. A reverse-phase HPLC analysis was performed with an Agilent Zorbax SB-C18 column (4.6 mm × 500 mm; 5 μm silica particle size). The porphyrins were eluted isocratically with acetonitrile: *N*,*N*-dimethylformamide: pyridine: acetic acid (80:20:0.5:0.5, v/v/v/v) at a flow rate of 1 mL min^−1^ and a column temperature of 20 °C. For the second purification step, a normal-phase HPLC analysis was conducted with an Agilent Zorbax SIL column (4.6 mm × 500 mm; 5 μm silica particle size). The mobile phase for the isocratic analysis was *n*-hexane: dichloromethane: *N*,*N*-dimethylformamide: pyridine: acetic acid (87:10:3:0.5:0.5, v/v/v/v/v) at a flow rate of 1 mL min^−1^ and a column temperature of 40 °C

The measurement of the stable nitrogen isotopic compositions of the porphyrins was similar to the measurement of the chloropigments. To obtain enough samples for isotopic measurements, the purified peaks were combined for each sample. Based on repeated measurements of our laboratory standards, the analytical error was estimated to be within 0.5‰ (±1σ).

The metal chelated in the porphyrins was determined with a quadrupole ICP-MS (iCAP Qc, Thermo Scientific, Bremen, Germany) using the metals standard solution VII (Kanto Chemical Co. Inc., Tokyo, Japan). The purified porphyrins were combined and reacted with 3 M HNO_3_ (high-purity TAMAPURE AA-100 reagents; Tama Chemical, Kanagawa, Japan) for 2 h under 130 °C to release the chelated metal. The relative standard deviations based on the replicate measurements of the sample were within 2.5% for the monitored elements (±2σ).

## Supplementary information

Supplementary materials

## Data Availability

All the data reported in this article are available from the corresponding author.
