# Supplementary material for: Efficient recycling of nutrients in modern and past hypersaline environments
Source: Sci Rep. 2019 Mar 6;9:3718. doi: 10.1038/s41598-019-40174-9 (PMC6403304; doi:10.1038/s41598-019-40174-9)
Supplement: Supplementary file 1 — Supplementary materials [file 41598_2019_40174_MOESM1_ESM.docx]

*Scientific Reports*

Supporting Information for

**Efficient recycling of nutrients in modern and past hypersaline environments**

Y. Isaji^1*^, H. Kawahata^2^, N. O. Ogawa^1^, J. Kuroda^2^, T. Yoshimura^1^, F. J. Jiménez-Espejo^1^, A. Makabe^3^, T. Shibuya^4^, S. Lugli^5^, A. Santulli^6^, V. Manzi^7^, M. Roveri^7^, and N. Ohkouchi^1^

^1^Department of Biogeochemistry, Japan Agency for Marine-Earth Science and Technology, ^2^Atmosphere and Ocean Research Institute, University of Tokyo, ^3^Project Team for Development of New-generation Research Protocols for Submarine Resources, Japan Agency for Marine-Earth Science and Technology, ^4^Department of Subsurface Geobiological Analysis and Research, Japan Agency for Marine-Earth Science and Technology, ^5^Dipartimento di Scienze Chimiche e Geologiche, Università degli Studi di Modena e Reggio Emilia, ^6^Istituto di Biologia Marina, Consorzio Universitario della Provincia di Trapani, ^7^Dipartimento di Scienze Chimiche, della Vita e della Sostenibilità Ambientale, University of Parma

**Contents**

Table S1

Table S2

Figure S1

Figure S2

Figure S3

Table S1 **Concentrations and δ^15^N values of nitrate and ammonium in the surface brine and porewater of the solar salterns in Trapani, Sicily, Italy.** The degree of evaporation is calculated from the magnesium concentration (DE_Mg_). Numbers in the sample names refer to the individual ponds at the salterns. CU, Culcasi; SS, Sosalt; CH, Chiusicella.

| Year | Sample | Pond type | Sample type | Salinity | DE_Mg_ | Nitrate conc. (µM) | Nitrate δ^15^N (‰) | Ammonium conc. (µM) | Ammonium δ^15^N (‰) |
| --- | --- | --- | --- | --- | --- | --- | --- | --- | --- |
| 2015 | CU-0 | Seawater |  | 38.2 | 1.0 | 2.2 | 1.9 | 12.0 | 27.6 |
|  | CU-1 | Carbonate | Surface brine | 96.8 | 2.7 | 0.8 | 3.8 | 17.0 | 3.5 |
|  | CU-2 |  |  | 126.0 | 3.4 | 5.3 | 1.2 | 23.6 | 11.8 |
|  | SS-3 |  |  | 151.0 | 4.7 | 5.0 | 1.2 | 18.4 | 22.7 |
|  | SS-1 | Gypsum |  | 159.1 | 4.9 | 5.4 | 1.3 | 19.1 | 21.8 |
|  | SS-2 |  |  | 179.6 | 5.8 | 5.6 | 1.4 | 20.6 | 20.9 |
|  | CH-1 |  |  |  | 11.9 | 13.9 | 0.0 | 38.3 | 25.0 |
|  | SS-4 | Halite |  |  | 11.2 | 8.6 | −0.3 | 25.3 | 22.6 |
|  | CU-5 |  |  |  | 22.0 | 30.9 | 0.5 | 55.6 | 34.0 |
| 2016 | CU-6 | Carbonate | Surface brine | 78.1 | 2.2 | 1.1 | −9.2 | 2.3 | − |
|  |  |  | Porewater |  |  | 0.6 | 7.9 | 334.3 | 6.5 |
|  | CU-7 |  | Surface brine | 108.4 | 3.1 | 0.5 | −7.0 | 0.4 | − |
|  |  |  | Porewater |  |  | 0.2 | 1.9 | 558.7 | 7.2 |
|  | CU-8 |  | Surface brine | 146.2 | 4.6 | 0.4 | −8.4 | 1.1 | − |
|  |  |  | Porewater |  |  | 0.2 | 7.6 | 315.4 | 6.6 |

Table S2 **δ^15^N of the bulk deposits and chloropigments, and the estimated source phototrophs inhabiting the microbial mats in the solar salterns in Trapani, Sicily, Italy, and the δ^15^N of the bulk sediments and porphyrins purified from the mud–anhydrite layer of Unit C (“Church” and “Rosone” sections) of the Realmonte salt mine.** The δ^15^N values of the cyanobacteria and purple sulfur bacteria are estimated from those of chlorophyll *a* and bacteriochlorophyll *a*, respectively, assuming that chlorophyll *a* is enriched in ^15^N by 9.8 ± 1.8‰ (1σ) and bacteriochlorophyll *a* depleted by 4.8 ± 1.4‰ (1σ) relative to the source phototrophs^1–3^. CU, Culcasi; SS, Sosalt; CH, Chiusicella; Chl *a*, chlorophyll *a*; BChl *a*, bacteriochlorophyll *a*.

| Year | Sample | Pond type | Layer | Bulk deposit δ^15^N (‰) | Compound | Pigment δ^15^N (‰) | Estimated  cell δ^15^N (‰) |
| --- | --- | --- | --- | --- | --- | --- | --- |
| 2015 | CU-1 | Carbonate | Top slimy | 1.9 | Chl *a* | 8.8 | −1.0 |
|  |  |  |  |  | BChl *a* | −0.4 | 4.4 |
|  |  |  | Black | 3.8 |  |  |  |
|  | SS-3 | Carbonate | Top slimy | 1.0 | Chl *a* | 13.1 | 3.3 |
|  |  |  | Black | 4.8 |  |  |  |
|  | SS-1 | Gypsum | Yellowish | 0.7 | Chl *a* | 12.7 | 2.9 |
|  |  |  | Green | 0.5 | Chl *a* | 17.2 | 7.4 |
|  |  |  | Pink | 2.6 | BChl *a* | −1.8 | 3.0 |
|  |  |  | Black | 4.6 |  |  |  |
|  | CH-1 | Gypsum | Yellowish | 3.4 | Chl *a* | 8.5 | −1.3 |
|  |  |  | Green | 4.8 | Chl *a* | 19.4 | 9.6 |
|  |  |  | Pink | 2.0 | BChl *a* | 0.1 | 4.9 |
|  |  |  | Black | 4.5 |  |  |  |
| 2016 | CU-6 | Carbonate | Top slimy |  | Chl *a* | 17.6 | 7.8 |
|  |  |  |  |  | BChl *a* | −2.2 | 2.6 |
|  |  |  | Black | 2.6 |  |  |  |
|  | CU-7 | Carbonate | Top slimy |  | Chl *a* | 12.0 | 2.2 |
|  |  |  |  |  | BChl *a* | −5.5 | −0.7 |
|  |  |  | Black | 2.9 |  |  |  |
|  | CU-8 | Carbonate | Top slimy |  | Chl *a* | 13.0 | 3.2 |
|  |  |  |  |  | BChl *a* | −8.1 | −3.3 |
|  |  |  | Black | 4.4 |  |  |  |
|  | Messinian sediment | | “Church” | 11.0 ± 0.42 (n = 2)* | Porphyrins | 17.2 |  |
|  |  | | “Rosone” | 6.1 ± 0.48 (n = 3)* | Porphyrins | 21.7 |  |

*Standard deviations (1σ) were determined with replicate measurements (n).


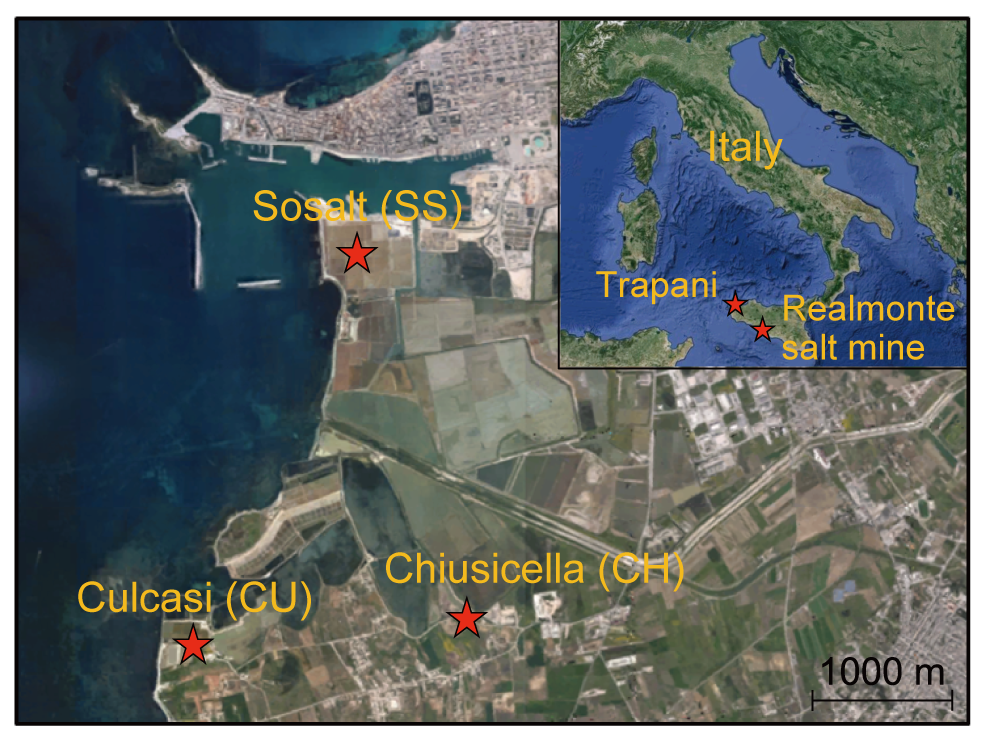


Fig. S1 **Sampling locations**. Map showing the locations of the three solar salterns in Trapani, Sicily, Italy, investigated in this study. Sediments deposited during the peak of the Messinian Salinity Crisis were collected from the Realmonte salt mine in Sicily, Italy. Modified from Isaji et al. (2017)^23^.

Fig. S2 **Estimates of the nitrogen isotopic fractionation factors during the assimilation of ammonium by purple sulfur bacteria (ε_PSB_) in ponds CU-6, -7, and -8.** The δ^15^N curves of purple sulfur bacteria (red lines) and the remaining ammonium (blue lines) as a function of the proportion of unutilized ammonium (*f*) were calculated for different ε_PSB_, assuming a Rayleigh distillation model, from the measured δ^15^N values of porewater ammonium (Table S1). Assuming that the amounts of ammonium assimilated by purple sulfur bacteria and cyanobacteria are comparable, as implied from the similar concentrations of chlorophyll *a* and bacteriochlorophyll *a* in each mat (i.e., *f* ranging around 0.5), the δ^15^N values of the purple sulfur bacteria (δ^15^N_PSB_) estimated from those of bacteriochlorophyll *a* give a rough estimate of the probable range of ε_PSB_, as well as the δ^15^N of the remaining ammonium (δ^15^N_NH4+_).


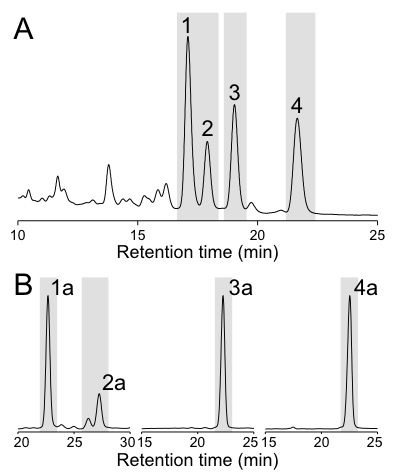


Fig. S3 **Chromatograms of the HPLC analysis of porphyrins.** (A) Reverse-phase HPLC chromatogram. Shaded peaks were collected for further purification. (B) Normal-phase HPLC chromatograms. Shaded peaks were collected and combined for measurement of their nitrogen isotopic compositions. The polarity of the extracted porphyrins on the silica-gel column, the photoabsorption spectra of the purified peaks (Soret band: 396–403 nm; Q-band: 559–565 nm), and the analysis with an inductively coupled plasma mass spectrometer conclusively determined these compounds as Fe-porphyrins.

**References**

1 Higgins, M. B. *et al.* Paleoenvironmental implications of taxonomic variation among δ^15^N values of chloropigments. *Geochim. Cosmochim. Acta* **75**, 7351–7363 (2011).

2 Ohkouchi, N., Kashiyama, Y., Kuroda, J., Ogawa, N. O. & Kitazato, H. The importance of diazotrophic cyanobacteria as primary producers during Cretaceous Oceanic Anoxic Event 2. *Biogeosciences* **3**, 467–478 (2006).

3 Sachs, J. P., Repeta, D. J. & Goericke, R. Nitrogen and carbon isotopic ratios of chlorophyll from marine phytoplankton. *Geochim. Cosmochim. Acta* **63**, 1431–1441 (1999).

4 Isaji, Y. *et al.* Biological and physical modification of carbonate system parameters along the salinity gradient in shallow hypersaline solar salterns in Trapani, Italy. *Geochim. Cosmochim. Acta* **208**, 354–367 (2017).
